# Should people with severe mental illness be prioritized for the COVID-19 vaccination?

**DOI:** 10.7150/ijbs.57750

**Published:** 2021-04-10

**Authors:** Yuan Yang, Wen Li, Qinge Zhang, Ling Zhang, Teris Cheung, Chee H Ng, Yu-Tao Xiang

**Affiliations:** 1 Unit of Psychiatry, Department of Public Health and Medicinal Administration, & Institute of Translational Medicine, Faculty of Health Sciences, University of Macau, Macao SAR, China.; 2 Centre for Cognitive and Brain Sciences, University of Macau, Macao SAR, China.; 3 Institute of Advanced Studies in Humanities and Social Sciences, University of Macau, Macao SAR, China.; 4 The National Clinical Research Center for Mental Disorders & Beijing Key Laboratory of Mental Disorders, Beijing Anding Hospital & the Advanced Innovation Center for Human Brain Protection, Capital Medical University, Beijing, China.; 5 School of Nursing, Hong Kong Polytechnic University, Hong Kong SAR, China.; 6 Department of Psychiatry, The Melbourne Clinic and St Vincent's Hospital, University of Melbourne, Richmond, Victoria, Australia.

**Keywords:** COVID-19, severe mental illness, vaccination

## Abstract

The coronavirus disease-19 (COVID-19) has spread throughout the world, affecting many vulnerable populations including patients with severe mental illness (SMI). Recent studies have found that patients with SMI compared to the general population could have a greater risk of morbidity and mortality from COVID-19 due to cognitive impairment, poor awareness of risk, and difficulties in complying with infection control measures. Although some researchers have suggested that patients with SMI should be prioritized for COVID-19 vaccination to reduce the risk of infection, this issue remains controversial.

The coronavirus disease-19 (COVID-19) has spread around the world, adversely affecting many vulnerable populations including patients with severe mental illness (SMI). Recent studies in some countries indicated that compared to the general population, patients with SMI are at a greater risk of morbidity and mortality from COVID-19 due to cognitive impairment, poor awareness of risk, and difficulties in complying with infection control measures 1. A recent retrospective cohort study conducted in the USA found a bidirectional association between psychiatric disorders and the occurrence of COVID-19; i.e., diagnosis of a psychiatric disorder might be an independent contributor to the occurrence of COVID-19, and vice versa 2. Mental illness is a major health challenge and also a leading cause of disability in many countries. Hence, researchers recently suggested that people with SMI should be prioritized for COVID-19 vaccination when available to reduce the risk of infection 1. The WHO has endorsed three principles regarding the prioritization of COVID-19 vaccines: 1) protect and promote human well-being: minimize harm and maximize benefits; 2) reciprocity: advocates prioritizing those individuals and groups that may bear significant additional risks and burden of COVID-19; and 3) equity: recognize and treat all human beings as having equal moral status 3. However, the WHO has not provided specific guidelines for patients with SMI alone. It remains debatable whether patients with SMI should be prioritized for COVID-19 vaccines unless they also have other significant risks and burden of COVID-19.

In the early stage of the COVID-19 pandemic, hundreds of patients with SMI were infected with COVID-19 in China. Later, the pandemic was rapidly brought under control in China by mid-March, 2020 and no further infections in SMI patients were reported. Conventional public health measures were strictly applied to SMI patients, such as social distancing, mask wearing, hand hygiene, setting up emergency infectious units and quarantine facilities, and isolating suspected and confirmed cases 4.

The successful measures implemented in China have demonstrated that the COVID-19 pandemic could be well controlled with conventional public health measures even among SMI patients. Hence, in China patients with SMI may not have a high infection risk or mortality risk due to COVID-19. Although the forthcoming COVID-19 vaccines may further reduce the risk of future waves of the pandemic, prioritizing COVID-19 vaccines for patients with SMI should be implemented with caution due to the following reasons.

First, the efficacy and safety of the COVID-19 vaccine have not been fully determined when the earliest batch of vaccine was released in early 2021. As of the end of October, 2020, more than 100 clinical trials on various COVID-19 vaccine types have been conducted, such as inactivated vaccine, adenovirus vector vaccine, attenuated influenza virus vector vaccine, and nucleic acid vaccine, all with varying strengths and shortfalls 5. Notably, due to the relatively short vaccine development period, the long-term safety of all COVID-19 vaccines remains unclear.

In addition, as an RNA virus, the Severe Acute Respiratory Syndrome Coronavirus 2 (SARS-CoV-2) has a complex molecular machinery resulting in a substantial number of mutations; therefore, the protective role and pathogenic aspects of the new COVID-19 vaccines are uncertain. Recent studies found that certain COVID-19 vaccines were associated with mild-to-moderate side effects, such as fatigue, headache, chills, muscle pain and high fevers, while coronavirus antibodies in human body tend to decline over time 6. Of note, immune system dysfunction is common in patients with SMI; for instance, previous studies have found that there is a positive link between autoimmune diseases and psychotic disorders 7. Giving the uncertainty of efficacy, safety, immunogenicity and duration of protection of the COVID-19 vaccine, certain high-risk subpopulations, such as patients with SMI, may not be the most suitable population to be prioritized for the vaccines. Another concern is that many SMI patients who receive the vaccine may incorrectly assume that they are completely immune and may therefore neglect to comply with preventive measures, which could in turn increase the risk of infection.

Second, many patients with SMI have impaired insight and decision-making capacity, and may be unable to provide informed consent to receive newly developed COVID-19 vaccines with controversial long-term efficacy and safety 8. Indeed, many people in certain countries have refused the COVID-19 vaccines due to fear of side effects and unknown long-term effects. Recently, a survey found that the acceptance rates of a COVID-19 vaccine varied greatly in the general population across countries, ranging from only 55% in Russia, and almost 90% in China 9. Therefore, informed consent before receiving the COVID-19 vaccines is an important ethical issue and it remains questionable whether SMI patients with impaired insight and decision-making capacity can provide informed consent to accepting the COVID-19 vaccination. Furthermore, certain psychiatric symptoms, such as hallucinations, delusions and disorganized behavior, could mask potential adverse reactions caused by the vaccines, which may compromise their safety.

Third, as most, if not all, clinical trials on COVID-19 vaccines have been conducted in strictly recruited healthy adults 10, the possibility of both lower efficacy and more side effects of COVID-19 vaccines in patients with SMI have not been examined. Given the common immune dysfunctions and physical co-morbidities in SMI patients compared to the general population 1, any newly developed vaccine should be given to this population with caution. More data on the efficacy and safety of COVID-19 vaccines in SMI patients are urgently needed.

In conclusion, the development of COVID-19 vaccines is a milestone in combating the pandemic, but due to the above-mentioned reasons, prioritizing COVID-19 vaccine for patients with SMI should be implemented with caution. In implementing a COVID-19 vaccine programme, selection of priority subpopulations should be considered carefully. Certain high-risk vulnerable populations, such as SMI patients, may not be suitable to be prioritized for the COVID-19 vaccines.
